# Structure of the hDmc1-ssDNA Filament Reveals the Principles of Its Architecture

**DOI:** 10.1371/journal.pone.0008586

**Published:** 2010-01-06

**Authors:** Andrei L. Okorokov, Yuriy L. Chaban, Dmitry V. Bugreev, Julie Hodgkinson, Alexander V. Mazin, Elena V. Orlova

**Affiliations:** 1 Wolfson Institute for Biomedical Research, University College London, Gower Street, London, United Kingdom; 2 School of Crystallography, Birkbeck College, Malet Street, London, United Kingdom; 3 Department of Biochemistry and Molecular Biology, Drexel University College of Medicine, Philadelphia, Pennsylvania, United States of America; 4 Institute of Chemical Biology and Fundamental Medicine, Siberian Branch of Russian Academy of Science, Novosibirsk, Russia; German Cancer Research Center, Germany

## Abstract

In eukaryotes, meiotic recombination is a major source of genetic diversity, but its defects in humans lead to abnormalities such as Down's, Klinefelter's and other syndromes. Human Dmc1 (hDmc1), a RecA/Rad51 homologue, is a recombinase that plays a crucial role in faithful chromosome segregation during meiosis. The initial step of homologous recombination occurs when hDmc1 forms a filament on single-stranded (ss) DNA. However the structure of this presynaptic complex filament for hDmc1 remains unknown. To compare hDmc1-ssDNA complexes to those known for the RecA/Rad51 family we have obtained electron microscopy (EM) structures of hDmc1-ssDNA nucleoprotein filaments using single particle approach. The EM maps were analysed by docking crystal structures of Dmc1, Rad51, RadA, RecA and DNA. To fully characterise hDmc1-DNA complexes we have analysed their organisation in the presence of Ca^2+^, Mg^2+^, ATP, AMP-PNP, ssDNA and dsDNA. The 3D EM structures of the hDmc1-ssDNA filaments allowed us to elucidate the principles of their internal architecture. Similar to the RecA/Rad51 family, hDmc1 forms helical filaments on ssDNA in two states: extended (active) and compressed (inactive). However, in contrast to the RecA/Rad51 family, and the recently reported structure of hDmc1-double stranded (ds) DNA nucleoprotein filaments, the extended (active) state of the hDmc1 filament formed on ssDNA has nine protomers per helical turn, instead of the conventional six, resulting in one protomer covering two nucleotides instead of three. The control reconstruction of the hDmc1-dsDNA filament revealed 6.4 protein subunits per helical turn indicating that the filament organisation varies depending on the DNA templates. Our structural analysis has also revealed that the N-terminal domain of hDmc1 accomplishes its important role in complex formation through domain swapping between adjacent protomers, thus providing a mechanistic basis for coordinated action of hDmc1 protomers during meiotic recombination.

## Introduction

In eukaryotes, homologous recombination (HR) is essential for the accurate segregation of homologous chromosomes during meiosis [1]–[3] and for the repair of DNA double-strand breaks (DSB) [4]–[6]. Mutations that impair meiotic recombination cause chromosome nondisjunctions leading to abnormalities such as Down's, Klinefelter's and other syndromes [7]. In addition, during meiosis, HR conducts out genetic exchanges between homologous chromosomes, producing a major source of genetic diversity [1]–[3].

Meiotic HR is initiated by DNA DSB introduced in chromosomes by a specialized enzyme, Spo11 [8]. DSB ends are subsequently processed by exonuclease(s) to yield 3′-overhanging ssDNA tails [3]. Members of the Rad51/RadA/RecA family are DNA strand-exchange proteins that bind these tails, seek out homologous DNA sequences and promote DNA strand exchange to generate joint molecules. In most eukaryotes including humans, this process is catalyzed by Dmc1 and Rad51 proteins, which are structural and functional homologues of bacterial and archaeal recombinases, RecA and RadA [3], [9], [10].

In contrast to the Rad51 recombinase that functions both in meiosis and somatic cell cycle during DSB repair, the Dmc1 protein family acts specifically during meiotic recombination [11]–[14]. In *S. cerevisiae*, the *dmc1* mutant shows almost complete absence of meiotic recombination [11], [15], [16]. In mice, the *DMC1* ^−/−^ knockouts were shown to be sterile whilst a hypomorphic mouse *DMC1^mei11^* allele (A272P) causes male-specific sterility [12], [13], [17].

A vast amount of structural data has been accumulated for RecA, RadA and Rad51 proteins, which were shown to form both rings and filaments without DNA [18]–[25]. The ssDNA-bound helical filaments of recombinases were studied by 3D electron microscopy and were shown to be present in two main states extended and compressed that represent active and inactive forms of the nucleoprotein complexes, respectively [26], [27].

Human Dmc1 is a protein of 340 amino acid residues that contains an N-terminal (1–81) and core ATPase (82–340) domain. Both domains are capable of binding DNA [18], [22], [28]. Recombinant hDmc1 produced in bacteria was shown to form hDmc1-dsDNA complexes that consist of stacked octameric rings when assembled in the presence of Mg^2+^ and ATP [14], [18], [22], [29]. The nucleotide cofactor ATP is required for the formation of a helical nucleoprotein hDmc1 filament on ssDNA, which subsequently promotes DNA strand exchange [30]. Recent studies have also shown that Ca^2+^ is an important factor in stimulation of the of helical filaments formation on ssDNA for both human and yeast Dmc1 leading to a major increase in the DNA strand exchange activity of hDmc1 [31]–[33].

The core domain of the hDmc1 protein has been crystallised in a DNA-free form, showing a structure similar to that of human and archaeal Rad51 proteins and providing information regarding the structural elements involved in DNA-binding [22]. Another recent report described the EM structure of the hDmc1-dsDNA filament showing that its parameters are very close to those of nucleoprotein filaments formed by other RecA family members [34]. However, the structural organisation of hDmc1-ssDNA complexes has remained unknown. To compare hDmc1-ssDNA complexes formed in the presence of ATP and Ca^2+^ with the RecA/RadA/Rad51 family nucleoprotein filaments we employed electron microscopy and single particle analysis. To fully characterise hDmc1-DNA complexes we have analysed their organisation under different conditions, e.g. in the presence of Ca^2+^, Mg^2+^, ATP, AMP-PNP, ssDNA and dsDNA. The obtained 3D EM maps of the ssDNA-hDmc1 nucleoprotein filaments, combined with fitting of atomic structures of the hDmc1 domains, allow us to outline for the first time the principles of the internal architecture of the hDmc1-ssDNA nucleoprotein filament that are most relevant to the presinaptic complex formation in the recombination process.

## Results

### Electron Microscopy and 3D Reconstruction of the hDmc1-ssDNA Filament

**Figure 1:**
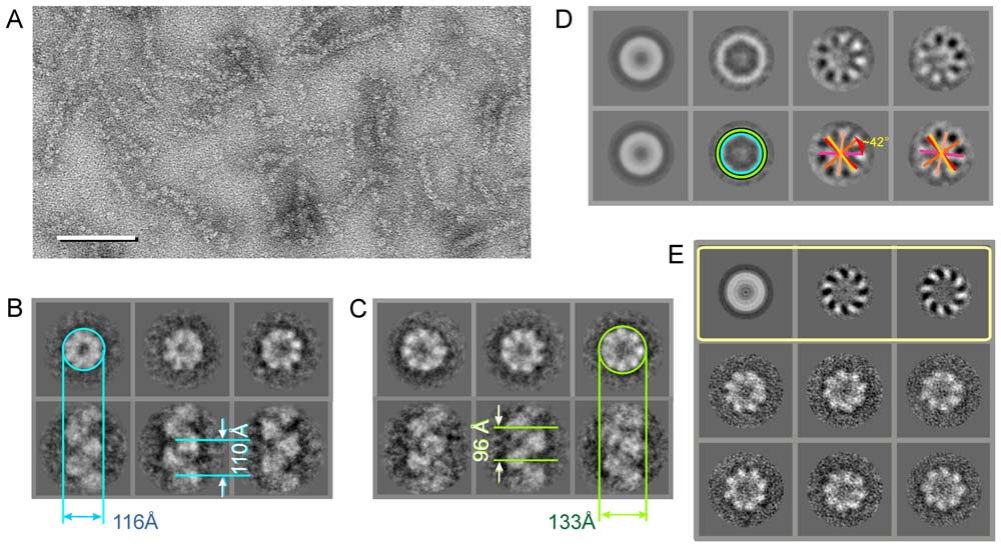
3D reconstruction of hDmc1-ssDNA filaments at a resolution of 16 Å. (A) Micrograph of stained hDmc1-ssDNA nucleoprotein filaments. The scale bar is 500Å. Ring-like paricles are indicated with yellow circles. (B) Representative class averages of the end and side views for the extended state are shown in upper and bottom panels respectively. (C) Representative class averages of the end and side views for the compressed state. Blue and green lines show sizes of filaments. The helical pitch sizes are ∼110Å and ∼96Å for extended and compressed states of the filament respectively. (D) Top row represents first four eigen images for the end views of hDmc1-ssDNA filaments. The second eigen image shows variations in diameters and the third and fourth images demonstrate the helical nature of the filaments. Bottom row shows the same eigen images as in top row where green and blue circles indicate diameters of the extended and compressed states respectively. An approximate protomer positions are indicated by red/yellow lines with an angle between them ∼42°. (E) The control sample of hDmc1 in the presence of Ca^2+^ and ATP, but in the absence of ssDNA consisted of hDmc1 rings only. The first three eigen images resulting from statistical analysis of these hDmc1 DNA-free ring particles are shown in the top row (in yellow frame). The characteristic class averages of rings are shown in the bottom two rows. Eight-fold rotational symmetry and handedness of rings are clearly visible.

**Figure 2:**
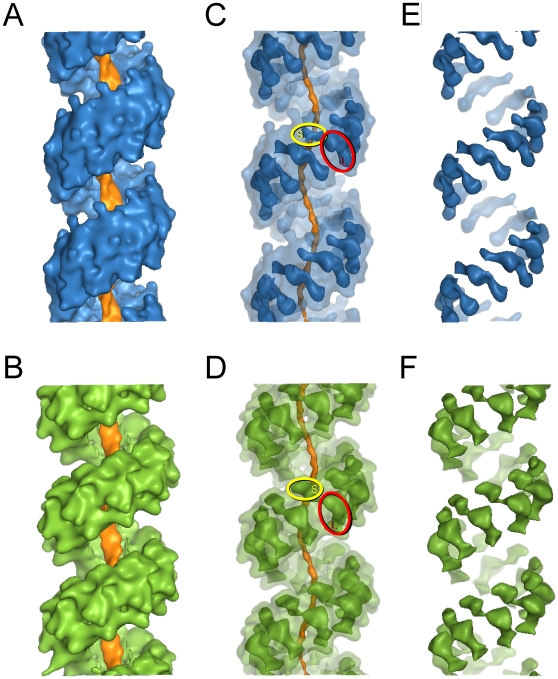
3D reconstruction of hDmc1-ssDNA filaments. (A) and (D) show views of the extended (blue) and compressed (green) state of the hDmc1-ssDNA filaments, respectively. The surface rendering is shown at the 1σ density threshold. Central density of the filament that corresponds to DNA is shown in orange at the 3σ density threshold. (B) and (E) show combined views with different density thresholds. The semi-transparent surface is shown at 1σ and the opaque view surface at 5.5σ. The internal density of the filament that corresponds to DNA is shown in orange at 4.5σ density threshold. Smaller and larger density regions of one protomer are marked as region S (in yellow circle) and region L (in red circle) respectively. (C) and (F) show views of the compressed (green) and extended (blue) state of the hDmc1-ssDNA filaments at 5.5σ density threshold.

Recombinant hDmc1 was expressed and purified to homogeneity from *E. coli* and analysed for its activity (Figure S1). Curved hDmc1-ssDNA filaments observed by electron microscopy (EM) prompted us to use the single particle approach (Figure 1A). Images of the hDmc1-ssDNA filament segments containing ∼2 helical turns were selected manually and image analysis was performed using IMAGIC-5 [35] (Methods). Statistical analysis of the images revealed two major states of hDmc1-ssDNA filaments that differ in diameter (Figure 1B, C, upper panels). Images corresponding to each state were processed as separate groups, while images of the distorted filaments were excluded from the reconstruction. Approximately 70% of the collected segments were of a smaller diameter, representing the extended state of the helical filament, while the other ∼25% of segments were wider in diameter and represented the compressed state of the hDmc1-ssDNA nucleoprotein complex. Small ring-like particles were selected for statistical analysis to determine if they may represent the end-views of short hDmc1-ssDNA filaments (Figure 1A, yellow circles). Resulting eigen images (see Methods) reveal a circular density gradient, in eigen images 3 and 4, confirming the helical nature of the particles (Figure 1D). As a control we analysed images obtained from the sample of hDmc1 in the presence of ATP and Ca^2+^, but in the absence of ssDNA. Under these conditions hDmc1 was present in the form of rings, yet no filaments or ring stacks were observed. The images of hDmc1 rings were subjected to the same procedure. These eigen images were strikingly different to those obtained for hDmc-ssDNA sample and typical for the rings with 8-fold symmetry (Figure 1E, upper row) [36]. Eigen images of the hDmc1-ssDNA end views allowed us to make the first assessment of the angles between protomers, and the pitch was estimated from the filament's images (Figure 1D). Helical parameters were refined by error minimisation between images and reconstruction projections (see methods). The 3D maps of both types of ssDNA-protein filaments were obtained at 16Å resolution at the 0.5 threshold of the Fourier shell correlation function (Methods). The extended state of the filament has pitch of ∼110Å and is ∼116Å in diameter. The compressed state of the hDmc1-ssDNA filament has a helical pitch size of ∼96Å and a diameter of ∼133Å. The handedness of both structures has been defined by fitting of the atomic models (see below). Both filaments have a continuous density that closely follows the central axis which can be attributed to the ssDNA (Figure 2A, B, C and D). The helical “thread” of DNA density inside of the nucleoprotein filaments has diameter of ∼18Å and 30Å in extended and compressed forms respectively. The extended and compressed states have protomer density orientated differently relative to the central axis. The protomers are clearly defined when analysed at high density thresholds (Figure 2C, E, D and F). Each protomer density appears to be composed of one larger and one smaller density regions, with the smaller region connected to the larger one by a linker. The small domain (S) appears to interact with the density of the large domain (L) of the adjacent protomer (Figure 2C and D). The two states of hDmc1-ssDNA filaments, extended and compressed, are most likely the consequence of hDmc1 protomers being bound to DNA at different stages of ATP hydrolysis similar to those of Rad51, RadA and RecA [26], [27], [29], [37]. For RecA and Rad51, compressed filaments are known to form in the presence of ADP; they are inactive because ADP does not support DNA-pairing activity of these proteins. The extended (active) filaments are formed in the presence of ATP. The ratio of active (70%) versus inactive (25%) in hDmc1-ssDNA filament state is presumably shifted towards the extended (active) state due to the presence of Ca^2+^, which slows down the ATP hydrolysis rate [31]. That has been confirmed by analysis of hDmc1-ssDNA filaments formed in the presence of Ca^2+^ and the non-hydrolysable ATP analogue AMP-PNP. The filaments formed under these conditions resemble the extended state of the hDmc1-ssDNA filaments by their respective pitch (∼100Å) and diameter (∼120Å) values (Figure S2A). The presence of Ca^2+^ was essential for filament formation, as previously reported [31]–[33]. Complexes of hDmc1 on ssDNA in the presence of ATP and Mg^2+^ hDmc1 formed stacks of rings on ssDNA template (Figure S2B and C) as described by other groups [14], [18], [31].

The overall appearance of the right-handed hDmc1-ssDNA filaments is similar to that observed for the Rad51- and RadA-DNA nucleoprotein complexes [27], [29], [37]. However, despite the previously reported conservation between known recombinases, our data demonstrate that hDmc1-ssDNA filaments have significantly larger diameter. Moreover, the density analysis clearly indicates that in contrast to the RecA/RadA/Rad51 family, the hDmc1-ssDNA filaments have ∼9 and ∼8.5 protomers per helical turn in the extended and compressed states respectively, instead of ∼6 found for other RecA family members. To verify the difference between hDmc1-ssDNA and RecA-ssDNA filaments we have analysed images of the latter (See Methods, Figure S3). The RecA-ssDNA filaments have pitch of ∼82Å and a diameter of ∼114Å, and thus correlate well to the compressed conformation of RecA-ssDNA filament, in agreement with results published by other groups [26], [27], [37], [38]. Our results were also different to the structure described for the hDmc1 nucloprotein filament formed on dsDNA [34]. To gain further insight in the origin of this difference we have analysed the structure of the hDmc1 filaments formed on dsDNA under conditions to those reported by Sheridan and co-authors [34]. The filaments formed under these conditions appeared more straight and rigid when compared with flexible hDmc1-ssDNA filaments. Although the pitch remained the same at ∼106 Å, these filaments were thinner (∼104Å diameter) than those formed on the ssDNA template (116Å diameter) (Figure S4).

### Domain Localisation

**Figure 3:**
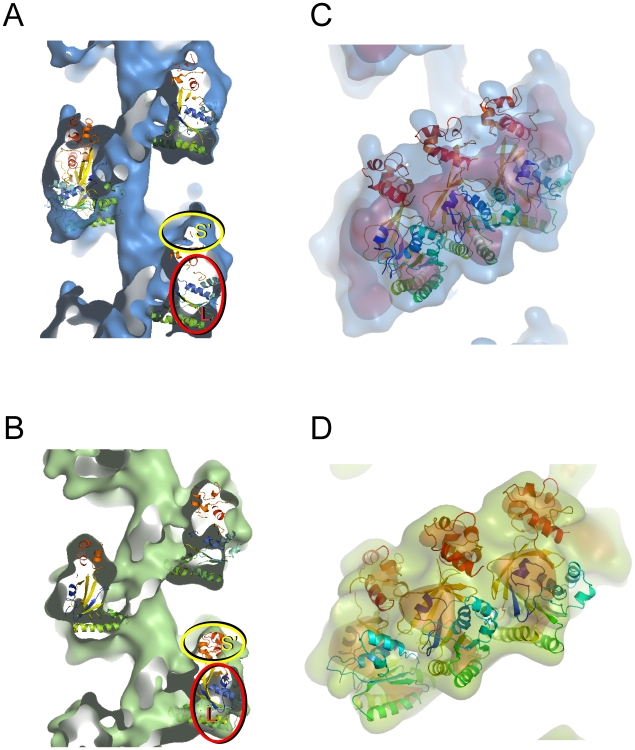
Docking of hDmc1 atomic coordinates into the 3D maps. hDmc1 core domain (PDB code 1V5W) [22] and the model of the N-terminal domain of hDmc1 (amino acid residues 1–81) based on the structure available for hRad51 (PDB code 1B22) were fitted into the 3D maps of both filaments. (A) and (B) show vertical 30Å thick central slabs of the density map with fitted hDmc1 protomers (N-terminus is in red). Surface rendering is shown at 1σ density threshold. The larger density regions are marked with L (in red circle) while S′ (in yellow circle) marks the smaller density region that belongs to the adjacent protomer. (C) and (D) show front slabs of the extended and compressed filaments with three adjacent hDmc1 protomers fitted into respective electron densities. The overall semi-transparent surface for both filaments is shown at 1σ and the inner surface at 3σ (orange in C and red in D).

Docking of atomic coordinates into the 3D maps of the two filament states was undertaken to analyse the relative positions of the N-terminal and core ATPase domains. As the N-terminal-domains of Rad51 and RadA are separated from their core domains by flexible interdomain linkers [19], [24] we opted to carry out simultaneous docking of two structures into the map: the hDmc1 core domain (PDB code 1V5W) [22] and a structural homology model of the N-terminal domain of hDmc1 (amino acid residues 1–81) based on the structure available for hRad51 (PDB code 1B22) [39]. An automated search using UROX software [40] and the UCSF Chimera package [41] was used to dock these structures into three adjacent protomers. The fitting procedure yielded unambiguous positions for the hDmc1 domains, in both extended and compressed states of the hDmc1-ssDNA filament (Figure 3).

The fitting positioned the hDmc1 N-terminal domain (coloured red) in the top of the protomer's density within the smaller region S (Figure 3), similar to that observed for RecA, Rad51 and RadA filaments [19], [26], [27], [29], [37]. According to our fitting, however, the N-terminal domain of hDmc1, within the smaller density region S, is positioned on the opposite side of the core domain when compared to the orientation reported for the Rad51 and RadA structures [19], [27], [37] (Figure S5). The difference is likely to be the result of the higher resolution of the 3D hDmc1 maps and the automated flexible fitting of the two separate domains as opposed to a docking of the full length protein structure as one rigid body. The relative orientation of the hDmc1 core domains after docking is consistent with that of hDmc1 protomers within the octameric ring obtained by X-ray crystallography [22]. The comparison of extended and compressed filaments shows that protomers are rotated 15° around the axis perpendicular to the filament axis, and 10° anti-clockwise in the plane parallel to the filament axis (Figure S6).

**Figure 4:**
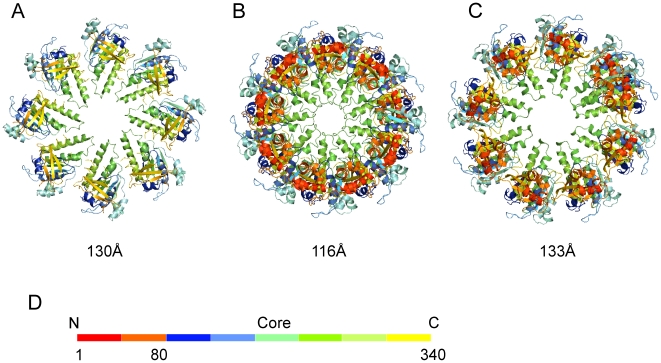
Comparison of the hDmc1 ring and filament assemblies. The octameric ring structure of hDmc1 (PDB code 1V5W) core domains (A) compared to end views of filament models in the extended and compressed states respectively (B and C). The respective diameters of filaments and ring are indicated beneath. (D) The colour-coded domain organisation of the hDmc1 protein sequence corresponds to the colours used in (A–C). “N” indicates N-terminal domain of hDmc1 (1–81aa) and “Core” indicates ATPase DNA-binding domain of hDmc1 (82–340aa).

Views along the rotation axes of the ring structure and of the ssDNA-bound hDmc1 filaments models (Figure 4A, B and C) show that the diameter of the compressed state of the hDmc1 filament (133Å) is very close to that of the ring (130Å, [22]). The diameter of the inner channels for the extended and compressed filament states and the octameric ring are 17.8Å 28.6Å and 27 Å, respectively.

### N-Terminal Domain Position

**Figure 5:**
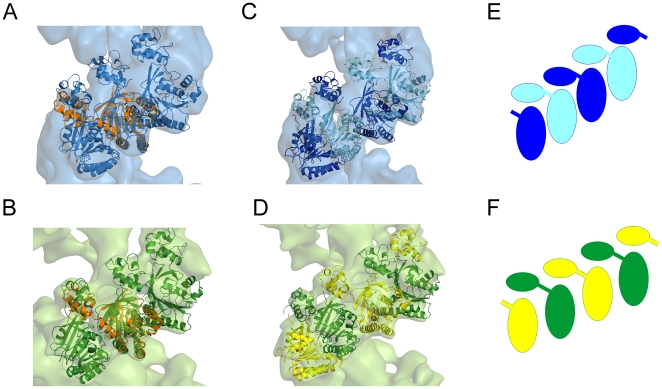
Internal organisation of the ssDNA-hDmc1 filament. Structure of the RadA filament protomer (in orange) is superimposed onto the (A) compressed and (B) extended states of the hDmc1-ssDNA filament. The protomers are aligned by their core domains. (C) and (D) show models for both filament states where the N-terminal domains are swapped between adjacent protomers. The N-terminal and core domains that belong to the same protomer are coloured accordingly, alternating yellow and green (C), and cyan and blue (D). The model is shown schematically in (E) and (F) for the extended and compressed filaments respectively.

Fitting the structure of the N-terminal domain into the filament reconstructions revealed an orientation where its C-terminal α-helix is juxtaposed against the N-terminal loop of the adjacent hDmc1 core domain of the protomer on the right side (Figure 5). The resulting combination of two domains shows remarkable similarity to the crystal structure of the RadA filament (2DFL) [24]. Indeed, when the RadA molecule (in orange) is superimposed onto this combination of hDmc1 domains it aligns rather well (Figure 5A and B). Such spatial organisation of two hDmc1 domains matches the protomer's density in both states of the filament. This strongly indicates that the protomers are not just neighbours within the filament, but are actively engaged with each other through N-terminal domain swapping (Figure 5C and D). The interaction between domains may be achieved in part via the outer basic patch of the core domain and the negative residues of the N-terminal domain which are in close proximity to each other. Such interactions would provide a linking chain-stabilising effect for the entire filament (Figure 5E and F).

**Figure 6:**
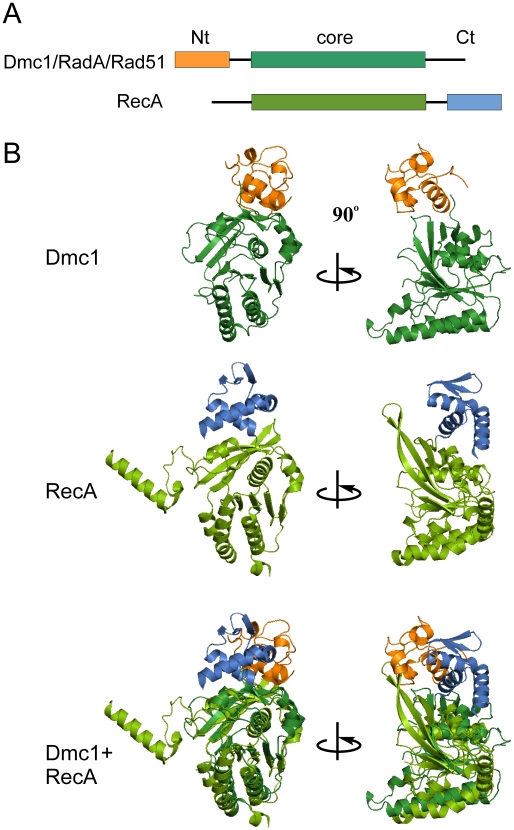
Comparison of hDmc1 protomer model with RecA structure. (A) Schematic representation of the domain organisation of the RecA/Rad51 recombinase family. (B) Structure of the hDmc1 protomer compared with the crystal structure of RecA. The N-terminal domain of hDmc1 is in orange, the C-terminal domain of RecA is in blue and the core domain is in green. The N-terminal domain and core domain of two adjacent hDmc1 protomers have the same relative positions as those held by the pair of the core and the C-terminal domain of one RecA protomer.

As with Rad51 and RadA, hDmc1 differs from RecA in that it has a ∼80 amino acid long N-terminal domain which has the ability to bind DNA [25], [33]. RecA has a similar functional domain at its C-terminus, whereas its N-terminal part, just prior to the core ATPase domain, is very short and is represented by a single α-helix [26] (Figure 6). The position of the N-terminal domain of one hDmc1 protomer in relation to that of the core ATPase domain closely resembles the spatial arrangement of the C-terminal and core domains of RecA (PDB codes 2REB and 1U94) [42], [43] (Figure 6). Comparison of the crystal structure of RecA with the model obtained by the docking of the hDmc1 domains into the 3D EM maps of the nucleoprotein filaments indicates that, despite the difference in the order of their domains within the protein sequence, the hDmc1 filament demonstrates a high degree of quaternary structural conservation with RecA.

### The ATPase Site and Protomers Interface

**Figure 7:**
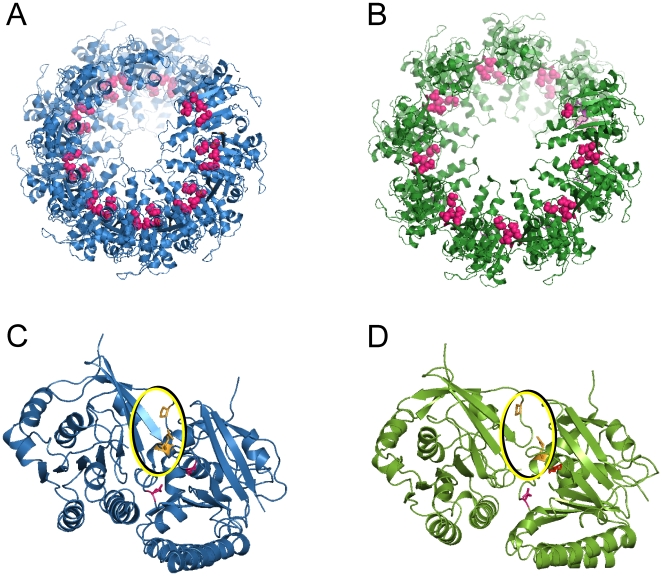
ATPase functional sites within the hDmc1-ssDNA filament. Amino acid residues of the Walker A and B motif residues are shown in pink for (A) the compressed and (B) the extended state of the hDmc1-ssDNA filaments. (C and D) depict the relative positions of the ATP-pocket residues in the compressed and the extended states respectively. Arg132 & Glu162 (the catalytic residues) are in pink, and Arg169 & Pro321 (the base tethering residues) are in the yellow circle and coloured orange.

The fitting of hDmc1 structures allowed us to analyse the location of the conserved amino acid residues that are involved either in DNA-binding or ATPase catalytic activity. The conserved amino acid residues of the ATPase catalytic site, such as Lys132 and Thr133 (Walker A motif) and Asp159 and Glu162 (Walker B motif), are located at the interface of adjacent molecules of hDmc1, similar to the locations described in the crystal structure of the octameric ring (Figure 7A and B, coloured pink). Amino acid residues implicated in ATP binding, namely Arg169 and Pro321, are separated in adjacent protomers by ∼13Å and ∼8.5 Å in extended and compressed states, respectively (Figure 7C and D). These values closely match the distances (19Å and 9.4Å) reported for the analogous residue pair Arg158 and Pro307 in *M. voltae* RadA [27]. This variation in the distances is consistent with the role these two residues play in ATP-base tethering [21]. They are expected to be brought into closer proximity in the active, extended state of the filament through changes induced upon binding to the ssDNA. Although the relative positions of protomers are similar in the octameric ring and ssDNA-bound filaments, the protomers in the ring are separated by larger distance. The distance between two base-tethering residues is ∼23Å, which is almost certainly too large to effectively accommodate the base in the ATP-binding pocket, explaining why ATP is not hydrolysed by the hDmc1 ring [31]. For comparison, the distance between these residues belonging to adjacent protomers in the hDmc1-dsDNA filament was ∼16Å, consistent with the fact that this type of nucleoprotein filament is recombinationaly unproductive.

### DNA-Binding Sites within the Filament

**Figure 8:**
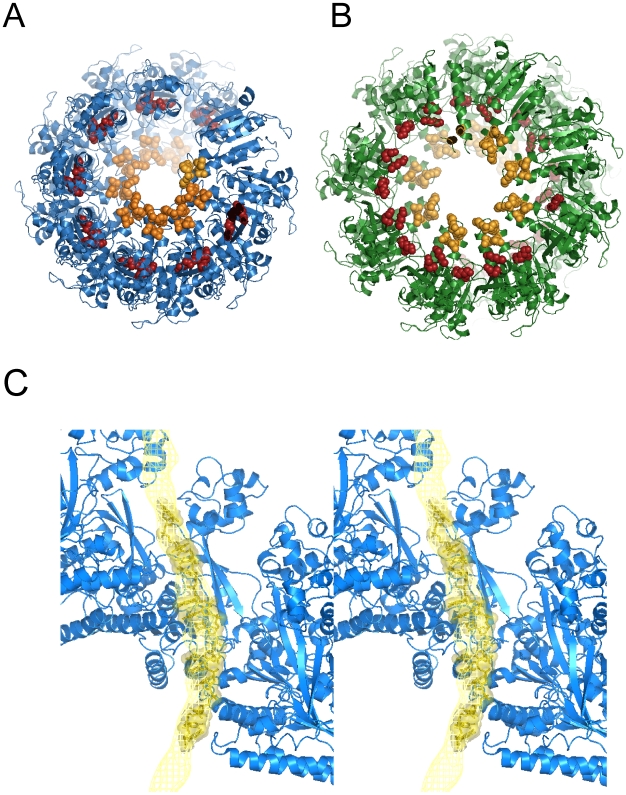
hDmc1-ssDNA interaction. The extended (A) and the compressed (B) state of the hDmc1-ssDNA filaments with amino acid residues of the “inner patch” of the core domain in orange and the basic patch residues of the N-terminal domain in red (C) Stereo view of hDmc1-ssDNA extended filament fitting model. 15 nucleotide long ssDNA (in dark yellow) from the RecA-ssDNA crystal structure (PDB ID: 3CMU) was fitted into the DNA density represented as a mesh (in yellow) at 3σ density threshold.

The electrostatic surface of fitted molecules for both extended and compressed filaments formed by ssDNA-bound hDmc1 protomers show predominantly negative charge on the outer surface and positive charge on the inner sides (not shown), which is consistent with the fact that ssDNA binds inside of the filament channel. From the crystal structure of the hDmc1 ring, positively-charged amino acid residues Arg230, Arg236 and Arg242, and the conserved Phe233 residue, form the so-called “inner” patch, which has been implicated in ssDNA-binding and tested by site-directed mutagenesis [22]. Consistent with the x-ray structure, all these residues are located within the inner channel of both extended and compressed hDmc1 filaments (Figure 8A and B, the residues coloured orange). Furthermore, the position of the N-terminal domain indicates that a group of positively-charged residues including Lys29, His30, Lys38, Lys41 and Lys63 forms another basic patch that faces the inner channel of the filament (Figure 8A and B, residues coloured dark red). Two residues from this group, Lys38 and Lys63, were reported to be involved in DNA-binding [25], [39].

To analyse the relative positions of hDmc1 domains with respect to ssDNA we have docked the structures of single-stranded DNAs from the RecA-ssDNA complexes. The tetranucleotide dTdAdCdG from NMR structure (PDB code 1EW1) [44] and 15-mer oligonucleotide from the crystal structure of RecA-ssDNA complex (PDB code 3CMU) [36] fitted well into the central density column of the 3D EM map of the extended filament. Indeed the docked 15-nucleotide long ssDNA follows the curvature of the central density thread. The docking of these structures has further validated our assignment of the central density in hDmc1 3D electron map to ssDNA (Figure 8C).

The fitted ssDNA threads along seven hDmc1 protomers, with each core domain of hDmc1 thus covering ∼2.1 nucleotides. This results in ∼19 nucleotides per helical pitch for 8.9 protomers per turn found in the extended state of the hDmc1-ssDNA filament. This is consistent with previous reports that in a recombinase-DNA complex the 5.1Å axial rise per base results in ∼19 nucleotides per turn [26], [29], [36], [45]–[47]. The inner positive patches of core domains appear to be in close proximity (3–4Å distance) with the phosphate backbone of the DNA strand (Figure 8C). At this level of resolution we are unable to say whether the specific nucleotide packing order observed for RecA-ssDNA complex is conserved here or not. However, given the fact that more protomers of hDmc1 are involved in covering the same length of ssDNA we would expect it to be different.

N-terminal domains do not appear to be in contact with the ssDNA; ∼20Å separate the DNA strand from the N-terminal basic residues (Figure 8C). It is likely that the main role of the N-terminal domain of hDmc1 in DNA-binding is utilized during synaptic complex formation when the N-terminus may bridge a connection between the filament and dsDNA. From our data hDmc1 appears to be the first reported DNA recombinase that uses ∼9 protomers per filament's turn to form a filament on ssDNA. This agrees with the measurements based on AFM analysis of Dmc1-ssDNA filaments that indicate the number of protomers per turn is higher than six and close to eight [32].

## Discussion

Similar to other recombinases, the hDmc1-ssDNA filaments described here are right-handed, which reflects the fact that dsDNA is itself a right-handed helix. The overall organisation of hDmc1-ssDNA nucleoprotein filaments in the presence of Ca^2+^ and ATP shows a high degree of evolutionary, structural and functional conservation with DNA-bound helical filaments of other human, archaeal, and bacterial recombinases [26], [27], [29], [37]. Two significant levels of evolutionary conservation in the structure-function relationship are observed in structures of hDmc1-ssDNA filaments. The first level of conservation appears to be in the spatial arrangement between the two domains of the recombinase molecules. Interestingly, the N-terminal domain and core domain of two adjacent hDmc1 protomers have the same relative positions as those held by the core and the C-terminal domain of one RecA protomer (Figure 6). This is consistent with the fact that the N-terminal domain of hDmc1, similar to that of Rad51 and RadA, and the C-terminal domain of RecA, have been previously implicated in DNA-binding, filament assembly and function [25], [26], [28], [39], [48]. It also supports the idea that the N-terminal domain of hDmc1 and the C-terminal domain of RecA share structural and functional roles [26]. Therefore, the second DNA-binding domain appears to be always positioned in the same manner with respect to the core domain, regardless of the domain order within the recombinase protein sequence.

The position of the smaller DNA-binding domain is linked to the second level of conservation, namely the N-terminal helix swapping between protomers. Three-dimensional domain swapping appears to be a common mechanism in allosteric coordination of oligomeric protein complexes [49]. The exchange of the N-terminal α-helices was previously reported for neighbouring protomeric subunits in RecA filaments [20], [37]. This principle of communication between protomers is conserved in hDmc1 helical filament organisation as the entire N-terminal domain of hDmc1 appears to be involved in domain exchange between adjacent molecules (Figure 5). The relative positions of the N-terminal and core domains of adjacent hDmc1 protomers provide a simple mechanism for the maintenance of filament architecture and could represent an optimal organisational system for the cooperative action of protomers during the DNA strand exchange process.

RecA, Rad51 and RadA were reported to have 6.2, 6.4 and 6.6 protomers per helical turn respectively [34], [38]. Our analysis of RecA-ssDNA helical filaments has also confirmed these helical parameters and the protomer per turn ratio is identical to the previous reports implying of 1 protomer covering 3 nucleotides (Figure S3). However, structures of hDmc1-ssDNA filaments shows a striking feature that distinguishes hDmc1 in that it employs 1 protomer to cover 2 nucleotides. Our findings are different from the recent report by Sheridan and coathors [34] that all recombinases share identical filament structure parameters.

One potential reason for the discrepancy between our and Sheridan's results could be in the DNA templates used for filament assembly. Our structural analysis of hDmc1-dsDNA filaments revealed that there are ∼6.4 protein subunits per helical turn (Figure S4) consistent with reconstruction of hDmc1-dsDNA filament by Sheridan and co-authors [34]. At the same time hDmc1-ssDNA filament structure has ∼9 protein subunits per helical turn. These results indicate that hDmc1 filaments formed on ssDNA and dsDNA templates vary in their architecture.

The hDmc1-ssDNA filaments we used for structural analysis match the biology of the first step of recombination process. The inner narrow channel of the extended filament corresponds to the diameter of ssDNA, while the wide inner channel of the compressed filament matches the diameter of the dsDNA. This data correlates to the standing model that the extended conformation corresponds to the initial active state of the presynaptic recombination complex. Thus we believe that our hDmc1-ssDNA structure reflects true biological and mechanistical properties of the presynaptic complex. The variation in the protomer number per helical turn between Dmc1 and the rest of the RecA family on ssDNA template may arise from the different recombinational events they are involved in, namely meiotic recombination and homologous repair respectively. For instance, Dmc1 filament architecture could reflect the fact that it deals with specific protein co-factors during meiotic recombination [3], [5].

In conclusion, we report the detailed molecular structure of the nucleoprotein filament formed on ssDNA by the human meiotic recombinase Dmc1. The 3D EM structure of the filament is consistent with previous biochemical and x-ray crystallography data, and allows us to elucidate the principles of its internal architecture. We show that there are ∼9 protomers per helical turn of the hDmc1-ssDNA filament in its active state, which might reflect the specific role of hDmc1 in meiosis. The filaments appear to be stabilized by N-terminal domain swapping between adjacent protomers. This provides a structural scaffold that allows for allosteric communication between hDmc1 protomers and their cooperative function during recombination.

## Materials and Methods

### Recombinant Proteins and Protein-ssDNA Complex Preparation

Human Dmc1 protein was purified as described previously [50]. ϕX174 ssDNA was purchased from Invitrogen. Nucleoprotein complexes were formed by incubating hDmc1 protein (10 µM) with ϕX174 ssDNA (15 µM) in buffer containing 25 mM Tris-acetate (pH 7.0), 2 mM ATP, 100 mM NaCl, 1 mM DTT, and 2 mM CaCl_2_ at 37°C for 30 min and then kept on ice for an additional 30 min [31]. Similar sample of hDmc1, but without ssDNA, was incubated as a control for visualisation of the hDmc1 DNA-free octameric rings. When Mg^2+^ was used instead of Ca^2+^ in control experiments it was at 2.5 mM concentration. When AMP-PNP (Sigma) was used it was at 2 mM concentration. Nucleoprotein complexes on dsDNA were formed by incubating hDmc1 protein (10 µM) with relaxed dsDNA in buffer containing 25 mM Tris-acetate (pH 7.0), 2 mM ATP, 100 mM NaCl, 1 mM DTT, and 2 mM CaCl_2_ at 37°C for 30 min and then kept on ice for an additional 30 min as described in [31]. Bacterial RecA was purchased from New England Biolabs (USA). Nucleoprotein complexes were prepared according to the manufacturer conditions by incubating RecA protein (10 µM) with ϕX174 ssDNA (15 µM) in buffer containing 50 mM Tris-HCl, (pH 7.5), 5 mM MgC1_2_, and 50 mM NaCl at 37°C for 30 min.

### Electron Microscopy, Image Processing

Samples for electron microscopy were stained with were 2% methylamine tungstate (MT), pH 6.8 (Nano-W, Nanoprobes Inc.) to keep pH close to physiological conditions. To test the effect of the MT staining on complex appearance samples were also stained with more conventional 2% uranyl acetate (UA) (pH∼4.5). All images were recorded on Kodak SO163 film using a FEI Tecnai T10 microscope in low-dose mode, operated at an accelerating voltage of 100 kV, and with a magnification of 44,000×. Negatives were developed in Kodak full strength developer D-19 for 12 min and their quality was assessed by optical diffraction. Micrographs were digitized on a Zeiss SCAI microdensitometer (Z/I Imaging) with a step size of 7 µm corresponding to a pixel size of 1.59Å. The contrast transfer function (CTF) of the microscope was estimated from incoherently averaged Fourier transforms of selected patches for each micrograph. Images were corrected for the CTF effects by phase-flipping procedure.

Segments of hDmc1-ssDNA filaments formed in the presence of ATP and Ca^2+^ (∼12,000 in MT and ∼2000 in UA) and RecA-ssDNA filaments (∼3000 in MT and ∼2000 in UA) were selected manually within frames of 160×160 pixels. No difference was detected between MT and UA staining during images processing. Control samples of hDmc1-ssDNA in the presence of AMP-PNP, hDmc1-ssDNA in the presence of Mg^2+^, hDmc1-dsDNA, and hDmc1 complexes formed in the absence of DNA were processed in similar fashion. Pre-processing of images included normalisation to the same standard deviation, with subsequent phase flipping and band-pass filtering to remove uneven background: the low-resolution cut-off was set to ∼100 Å with 10% remaining transmission of low frequency component; the high-resolution cut-off was ∼7Å. Image analysis was performed using IMAGIC-5 [36].

Filament segments were selected using only two constrains: (i) they had to be straight and (ii) the length of the segments should be at least 3 helical pitches. Distinct end views of the hDmc1-ssDNA complexes and hDmc1 rings formed in the absence of ssDNA were selected from the micrographs of MT stained specimens. End views of filaments (sample in the presence of ssDNA) and rings (sample without ssDNA) were subjected to statistical analysis, with each set processed separately [51]. The total sum (an average of all end-views images, sample with ssDNA) is seen as the first eigen image. While eigen image 2 demonstrates changes in the diameter [52], eigen images 3 and 4 reflect a non-integer number of subunits per turn (Figure 1D). The circular variations in the density distribution revealed in the third and fourth eigen images are related to the distribution of protomers in helices. Statistical analysis of the rings (hDmc1 without DNA) demonstrated that the number of protomers in them differs from that in the helices. The images of the rings did not reveal any variations in their sizes, and a pair of complementary eigenvectors confirmed 8-fold symmetry of the complexes (Figure 1E, top row). The double rings, where the percentage of tilted particle images was rather high (Figure S2D and E), did not show low frequency variations in eigen images since the double ring structure has an integer number of subunits.

Alignment and classification of hDmc1 filament segments were performed as described [51]. The multi-statistical analysis (MSA) of side views yielded defined classes, which revealed variations in pitch and diameter (Figure 1B and C, bottom panels). Preliminary reconstructions were obtained for both subsets by angular reconstitution [51] using short segments of several classes with good contrast, distinctive features such as polarity and separated subunits, and a few classes of hDmc1-ssDNA end-views [51]. The subsequent refinement was performed in a way similar to that described by Galkin and coathours [27]. The first stage of refinement of parameters was performed with increments of 3.2Å and 2° to find global minimum in the range of 35–48°. Five initial models obtained from the best classes were averaged and the central part of each model was symmetrised using helical parameters of the pitch (100 Å) and rotational angle between protomers (40°). Reprojections of the averaged model were used to refine alignment of all selected segment images. The next round of refinement was performed with increments of 0.5Å and 0.5° in the area of the established minimum (Figure S7). This resulted in two groups of parameters, which corresponded to extended and compressed states of the filament, which were processed independently. The extended state has pitch of 110 Å, diameter of ∼116 Å, and the angle between protomers is 40.5°. The compressed state of the filament has a pitch of 96 Å, diameter of 133 Å and the angle between protomers is 42.5°. The number of obtained classes was 550 for the extended formation and 250 for the compressed conformation, each containing ∼15 images per class. The final reconstructions of the hDmc1-ssDNA filament were calculated using 300 classes for the extended and 150 classes for the compressed state.

Similar procedure has been used for analysis of the RecA-ssDNA filament images. The starting search has been performed in the range of 40–70 degrees with a step of 3° followed by refinement with the found minima with 1° step. That resulted in the angle between protomers equal to ∼59°. The final reconstructions for RecA-ssDNA compressed filament was obtained from several best classes (Figure S3). For the hDmc1-dsDNA filament images the search has been performed in the range of 30–70 degrees with a step of 3° followed by refinement with the found minima with 1° step. That resulted in the angle between protomers equal to 56.5°. The final reconstructions for hDmc1-dsDNA complex was obtained using 50 best classes (Figure S4).

Orientations of class images were refined using the projection matching procedure in Spider [51]. 3D maps were calculated using the exact-filter back projection algorithm [50], [53]. Resolution of filament maps has been assessed using the 0.5 threshold of Fourier Shell Correlation function [54], [55] and corresponded to 16Å for hDmc1-ssDNA filaments and ∼27 Å for RecA-ssDNA filaments.

### Model Fitting

Secondary structure prediction was performed using Swiss-Model (http://swissmodel.expasy.org/) [56] and PHYRE (http://www.sbg.bio.ic.ac.uk/phyre/html/) from the Structural Bioinformatics Group, Imperial College, London. The best resulting model was built on the basis of the human Rad51 (PDB ID: 1b22) structure (40% of amino acid sequence identity) with E-value of 7.9e-14 and 100% of the estimated precision.

Domain fitting into the 3D map of hDmc1 was performed automatically using UROX (http://mem.ibs.fr/UROX/) [40] and by UCSF Chimera package from the Resource for Biocomputing, Visualization, and Informatics at the University of California, San Francisco (supported by NIH P41 RR-01081), (http://www.cgl.ucsf.edu/chimera/) [41]. Fitting of the atomic structure of RecA-ssDNA (PDB ID: 3CMU [38] into the 3D map of RecA-ssDNA was done manually in PyMol (http://pymol.sourceforge.net/) and Chimera.

Surface representations were generated using PyMOL and unless stated otherwise are displayed at a threshold level of 1σ corresponding to ∼100% of the expected mass (protein specific density is 0.84 kDa/Å^3^).

#### Accession code

The EM maps of human Dmc1 protein have been deposited in the macromolecular structure database (EBI) with accession number EMD-1492 and EMD-1493 for the extended and compressed filaments respectively.

## Supporting Information

Figure S1 Purified human Dmc1 efficiently catalyzes DNA strand exchange. A. Human Dmc1 was overexpressed in the E. coli strain BLR(DE3) (Novagen) as an N-terminal hexahistidine-tagged protein, purified and analyzed by electrophoresis in a 15% SDS-polyacrylamide gel. Extract from a 6 liter cell culture (lane 2) was subjected to ammonium sulfate precipitation (lane 3) and chromatographic fractionation on HisTrap HP (lane 4), HiTrap Heparin HP (lane 5), and Mono Q columns to purify Dmc1 to near homogeneity (5 ug in lane 6). Lane 1 shows migration of molecular weight markers. B - DNA strand exchange activity of purified human Dmc1 was tested in the D-loop assay according to the scheme. The red asterisk indicates 32P label at the 5′-end DNA. C - The products of DNA strand exchange were analyzed by electrophoresis in a 1% agarose gel. To form D-loops, 32P-labeled ssDNA; 5′-ACGCATCTGTGCGGTATTTCACACCGCATATGGTGCACTCTCAGTACA ATCTGCTCTGATGCCGCATAGTTAAGCCAGCCCCGACACCCG-3′; 3 uM, nucleotides) was preincubated with Dmc1 (in indicated concentration) in buffer containing 25 mM Tris acetate, pH 7.5, 2 mM ATP, 2 mM calcium chloride, 2 mM DTT, and BSA (100 ug/ml) for 15 min at 37 C. D-loop formation was initiated by addition of pUC19 scDNA (50 uM, nucleotides) followed by a 15-min incubation. In control (lane 1), storage buffer was added instead of Dmc1. The reactions were stopped by addition of 1.5% SDS and proteinase K (800 ug/ml), mixed with a 0.10 volume of loading buffer (70% glycerol, 0.1% bromophenol blue) and analyzed by electrophoresis. D - The data from panel (C) presented as a graph. μμμ
(2.10 MB TIF)

Figure S2 hDmc1 complexes assembled on ssDNA template at different conditions. A - Class averages of hDmc1 filaments formed on ssDNA template in the presence of AMP-PNP and Ca2+. B -Image of hDmc1 ring stacks formed on ssDNA in the presence of ATP and Mg2+. C - Class averages of hDmc1 ring stacks formed on ssDNA in the presence of ATP and Mg2+. D - Class averages of hDmc1 double rings formed on ssDNA in the presence of ATP and Mg2+. E - Eigen images from statistical analysis of the double rings shown in panel D.
(4.87 MB TIF)

Figure S3 Comparison of RecA and Dmc1 EM images. A - Side views representative of class averages. Upper panel and bottom panel show RecA and Dmc1 respectively both in the compressed state. B - Comparison of the side views: image of RecA has been aligned to the image of Dmc1 and subtracted from it. The differences between diameters and pitches of two filaments is clearly visible. Left panel shows images with sizes, right panel represent the same images not obscured by labels. C - 3D reconstruction of RecA demonstrates that the angle between protomers is ∼59°, resulting in 6.1 RecA protomers per helical turn. D - Central sections of the filament. Right panels for C and D show slabs of 3D EM density with fitted atomic structure of the RecA-ssDNA complex (PDB ID: 3CMU). Lines in pink indicate thickness and position of the slab.
(4.23 MB TIF)

Figure S4 hDmc1 filaments assembled on dsDNA template. A - Image of hDmc1-dsDNA complex demonstrates straight and rigid filaments. B - Class averages of hDmc1-dsDNA filaments. C - 3D map of the hDmc1-dsDNA filament. The angle between protomers is ∼56.5°, resulting in 6.4 protomers per helical turn. D - Slab sections of the hDmc1-dsDNA filament with docked model of dsDNA, hDmc1 core domain (PDB code 1V5W) and homology model of the N-terminal domain based on the structure of hRad51 (PDB code 1B22).
(4.54 MB TIF)

Figure S5 Position of the N-terminal domain of Dmc1 compared with known recombinase structures. Structure of the Dmc1 protomer compared with the crystal structures of RadA (2DFL), RecA (2REB) and Rad51 (1SZP). The N-terminal domains of Dmc1, RadA and Rad51, and the C-terminal domain of RecA are in orange. The core domains are shown in rainbow colouring with N-termini in blue and C-termini in red.
(1.23 MB TIF)

Figure S6 Core domain orientation changes between compressed and extended states of Dmc1-ssDNA filament. Protomer in the extended filament (in blue) is rotated 15° clockwise from its position in the compressed filament (in green) relative to the filament axis (in orange), left panel, and 10° anti-clockwise in the direction perpendicular to the filament axis, right panel.
(1.66 MB TIF)

Figure S7 Search for helical parameters of Dmc1-ssDNA filaments. It has been performed by error minimisation between images and their reprojections. This resulted in two groups of parameters, which corresponded to compressed and extended states of the filament. The compressed state of the filament has a pitch of 9.6 nm, diameter of 13.3 nm and the angle between protomers is 42.5°. The extended state has pitch of 11 nm, diameter of ∼11.6 nm, and the angle between protomers is 40.5°. The yellow arrows indicate positions of the global minima.
(5.40 MB TIF)
